# Reply to the ‘Comment on “Uncommon structural and bonding properties in Ag_16_B_4_O_10_” by A. Lobato, Miguel Á. Salvadó, and J. Manuel Recio, *Chem. Sci.*, 2021, 12, DOI: 10.1039/D1SC02152D

**DOI:** 10.1039/d1sc04494j

**Published:** 2021-09-29

**Authors:** Congling Yin, Ulrich Wedig, Martin Jansen

**Affiliations:** MOE Key Laboratory of New Processing Technology for Nonferrous Metal and Materials, Guangxi Key Laboratory of Optical and Electronic Materials and Devices, College of Materials Science and Engineering, Guilin University of Technology Guilin 541004 P. R. China; Max-Planck-Institut für Festkörperforschung Heisenbergstr. 1 70569 Stuttgart Germany m.jansen@fkf.mpg.de

## Abstract

Where are the excess electrons in Ag_16_B_4_O_10_?
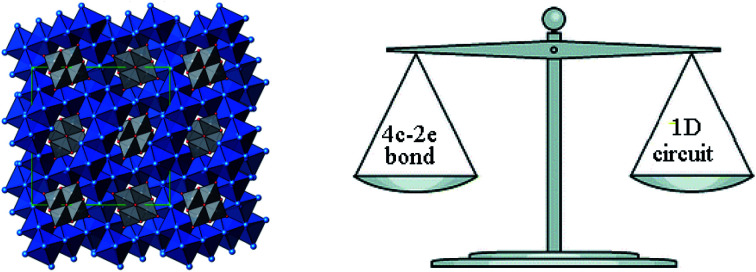

Ag_16_B_4_O_10_ features an exotic scheme of chemical bonding and extends the growing family of subvalent silver oxides. These findings constitute a new general and intrinsic facet of the chemistry of silver, which has not been fully understood, yet, and definitely deserves to be analysed from different perspectives. Against this background, we distinctly appreciate the efforts made by A. Lobato, Miguel Á. Salvadó, and J. Manuel Recio (LSR) in studying these phenomena at the example of the title compound.^1^ While the computational results presented in the Comment article well comply with those published in our original paper,^2^ the interpretations follow different routes. Whereas LSR focus on the analogy of pattern of the Electron Localization Function (ELF) in position space in the title compound with those found in elemental silver, we interpreted the electronic structure of Ag_16_B_4_O_10_, both in position and reciprocal space, also considering the interactions between cationic and anionic partial structures.

## Background

In structuring our discussion, we qualitatively rank the contributions of the different types of chemical bonding to the total cohesion energy of the title compound. The markedly largest share results from conventional covalent and ionic bonding, followed by much weaker unconventional d^10^–d^10^ interactions.^3^ The latter contribution is a crucial ingredient in the formation of the agglomerates of equally charged Ag^+^, which represent excisions of the ccp structure of elemental silver, even with respect to interatomic distances. These unique structural characteristics are evolving in silver-rich oxides already without presence of residual 5s electrons, *i.e.* in electron precise Ag^+^ compounds. Still, in an early review,^3^ the expectation was expressed that these features may enable existence of subvalent species: “The substructures thereby formed have empty s and p conduction bands, which can easily accommodate further electrons on reduction”. This assumption has become true, as meanwhile several such subvalent silver oxides have been discovered,^4–14^ in addition to previously confirmed Ag_2_F and Ag_3_O.††The references 3–5 given by LSR for evidencing early examples of subvalent silver are misleading. They rather refer to an unidentified material (1887) or to silver(i) complexes and salts with subvalent carbon oxides and ketene, respectively.^,^^15,16^

The most puristic manifestations of bonding interactions between presumably closed shell d^10^ species are found in the distorted hcp structures of elemental zinc and cadmium. Here, the set of commonly equal distances to the 12 nearest neighbours of an atom in an ideal hcp structure is conspicuously split into subsets of six substantially shorter in-plane and six longer out-of-plane separations. Density functional (DFT) calculations do not give an unequivocal picture of this anisotropy as the results strongly depend on the functionals used.^17^ By applying wavefunction based methods in the framework of the method of increments,^18,19^ the potential energy surface (PES) with respect to the lattice parameters was analysed.^20,21^ While the Hartree–Fock PES is overall repulsive, a structured PES consistent with the observed structures can only be obtained if the filled d-shells are included in the treatment of the dynamical electron correlation.

Such calculations, which are computationally highly demanding, have not yet been carried out for subvalent silver compounds, and a quantitative explanation for the d^10^–d^10^ bonding in silver-rich compounds, which on its turn would be a crucial prerequisite for rationalizing existence of the subvalent silver oxides under discussion, is still elusive. However, this issue is not the central subject of the comment by LSR, which focuses on the localisation of the residual 5s electrons, rendering silver subvalent, while contributing a smaller portion to the total of cohesion energy.

## The excess electrons in the silver partial structure of Ag_16_B_4_O_10_

In their analysis, LSR follow a particular line of reasoning, spotting the evolution of the electron localisation function (ELF) starting from the fcc elemental silver, moving *via* the silver sub-array as excised from Ag_16_B_4_O_10_ to the integral compound. The results on the two structures consisting of silver alone are illustrative as such, and underline the merits of this approach for visualising and interpreting the bonding in metals and intermetallics in real space.^22^ However, except for confirming that the excess electrons preferably accumulate in the tetrahedral voids (the sites of highest positive potential), the worth of these findings in understanding the bonding in Ag_16_B_4_O_10_ is but limited. At least from the Ag–O bond lengths, which indicate presence of strong, regular bonds between the silver and borate fragment structures, it is obvious that it is not possible to separate these structural parts without causing severe perturbations. Thus, the fragment silver structure cannot serve as a reliable reference for the same unit containing the embedded borate anion. For this reason, we focus in our reply on the electronic properties of the entire configuration Ag_16_B_4_O_10_. The computational results obtained in both studies comply satisfactorily. LSR do not state if they encountered a gapped band structure. In our PBEsol-GGA-calculations^23^ the gap is closed, although showing a minimum of the density of states (DOS) at the Fermi level. Only by applying a hybrid functional,^24^ a gap is opened. According to our experience, however, this issue is not crucial for the evaluation of the ELF. Importantly, in both studies^1,2^ the most populated ELF basins within the silver substructure are found at the same sites. Yet, the exegeses of the computational results are appreciably divergent.

One must keep in mind that the ELF is not an “observable” in terms of quantum chemistry, since there is no Hamiltonian which operating at an appropriate wave function would reproduce such features, and correspondingly there is no experimental tool available that would allow to directly validate such results. ELF does not prove the existence of electron pairs, bonds, or lone pairs. But there is a strong and appealing analogy between ELF attractors and basins, and classical Lewis structures, enabling to interpret the 3D-ELF in terms of a conceptual view of bonding. The analogy gets weaker in intermetallic compounds or in compounds including transition elements. Especially with late transition elements (like Ag) the values of the valence-(s,p)-attractors are much lower than the values of the d-attractors, see analysis by Kohout, Wagner and Grin.^25^ Clearly, interpretation of ELF features falls within the scope of chemical concepts.^26^ In our attempt to rationalise the at first glance puzzling experimental observations of an electron imprecise extended oxide to show semiconducting and diamagnetic responses, we assume that the eight excess electrons per formula unit will localise pairwise with opposite spin orientation. In consent with LSR, we regard the (B_4_O_10_)^8−^ anion as electron precise, consequently the excess electrons would be hosted by the silver partial structure classifying this compound as subvalent with respect to silver. For identifying possible localisations of such electron pairs, we inspected the silver part of the structure for short Ag–Ag separations, which might indicate presence of *e.g.* 2c–2e bonds, and performed band structure calculations along with an ELF analysis. As a result, we identified contracted tetrahedral subunits where 3 out of 6 Ag–Ag contacts along the edges of the tetrahedron are significantly shortened and were the ELF shows the highest value not associated to conventional bonds or lone pairs. Quite satisfactorily, the number of such building blocks per unit cell exactly corresponds to the number of electron pairs to be accommodated. Being aware that a partition of space, be it for structuring the electron density distribution or the ELF of a chemical entity, always suffers from arbitrariness, even if performed by applying a formal algorithm, we did not claim that the ELF contour drawn within the tetrahedra would comprise a complete pair of electrons with anti-parallel spins. With this respect LSR misinterpreted our statement that the excess electron pairs were related to the contracted Ag_4_ units.

LSR go beyond our interpretation by considering regions with lower ELF values, which indicate that the maxima are linked through ELF first-order saddle points or basin interconnecting points (bips) bringing about extended chemical entities, *i.e.*.superbasins. At this point, we are discussing the spatial extension of the localized electron pair. Unfortunately, there is no means of reliably validating which of the two views, assuming extended, metallic super basins or more localised electron pairs, would rather be appropriate in interpreting the bonding situation encountered.

In fact, we do not consider the extension of the localized electron pair as being pivotal to the physical properties and the stability of Ag_16_B_4_O_10_. The analysis of the electronic structure in reciprocal space shows that bands with notable Ag-s-character are found far apart from the Fermi level at −6 eV. Just below the Fermi level, the band structure as given in Fig 7 of ref. 2 shows a low DOS in a range of 1.4 eV. This is due to some bands with high dispersion. The electron density of these bands is mapped in Fig. 7b of ref. 2, illustrating the linear combination of Ag-d-orbitals and oxygen lone-pairs. This can be understood as a continuation of d^10^–d^10^ interactions in the silver partial structure to the lone-pairs of the B_4_O_10_^8−^ anion *via* dispersion interaction, stabilizing the whole structure and leading to the semiconducting behaviour of the compound.

**Fig. 1:**
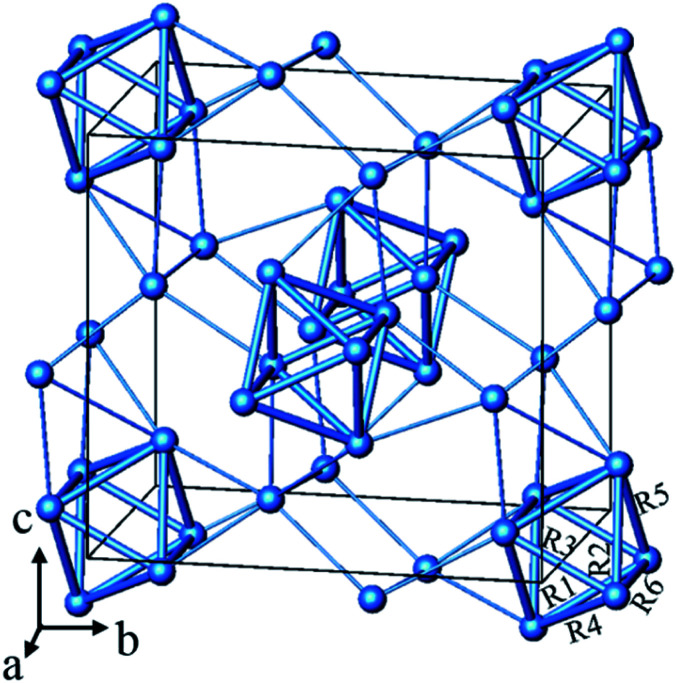
The Ag substructure of Ag_5_GeO_4_ consists of contracted Ag_6_ octahedra and “isolated” Ag cations. The d_Ag–Ag_ < 2.89 Å are shown as bold rods while the 2.89 Å < d_Ag–Ag_ < 3.50 Å are shown as thin rods. The Ag–Ag distances are R1 = 2.846 Å, R2 = 2.884 Å, R3 = 2.737 Å, R4 = 2.880 Å, R5 = 2.782 Å and R6 = 2.815 Å.

From the chemistry perspective of trying to define a generalizable “concept” for this particular type of bonding, one would compare with analogous compounds. In Fig. 1 we present the silver sub-structure of Ag_5_GeO_4_ (ref. 10 and 11) as an example, giving appreciable support to a situation of local bonding. Here again, the number of contracted polyhedra (octahedral Ag_6_ units) corresponds to the number of electron pairs to be accommodated, further the silver clusters and are not aggregated which rules out significant delocalisation.

## Concluding remarks

Manifestations of subvalent silver in extended solids can no longer be regarded as exotic singularities since in recent years a sizeable number of oxides displaying such characteristics have been reported.^4–16^ From the empirical point of view there are two particularly noteworthy issues: (1) subvalent silver has been encountered in combination with quite diverse cations, *e.g.* B, Si, Ge, Pb, Ni, Mn, Os or Pt, apparently without any systematic showing up, which nourishes the expectation that many more such candidates will be accessible; (2) opposite to common presumption, these oxides are strikingly stable in humid air and in particular against oxidising conditions, they even form by solid–state reactions applying elevated oxygen pressure. In a heuristic approach, one may separate the total of bonding interactions responsible for the very specific phenomena featured in conventional covalent and ionic contributions, and dispersive d^10^–d^10^ forces, superimposed by additional bonding provided by the excess electrons populating bonding 5s bands or local 5s/5p skeleton orbitals. While the first two contributions appear to be sufficient for forming the extended subarrays of Ag^+^, providing low lying, empty 5s states,^3^ suited to accommodate excess electrons, the third component is subordinate and just gently modulates the silver substructures as indicated by global or local contractions. The low bonding energy of the latter explains the wide phenomenological spread with respect to structure modulations and properties encountered. Thus, one finds extended silver subarrays *e.g.* of Ag^0.5+^ in Ag_2_NiO_2_^6,13,14^ or Ag_2_F,^15^ or locally shrank tetrahedral or octahedral units embedded in the silver substructure.^2,9–12^ Correspondingly, metallic conduction and semi-conducting properties, respectively, have been found.

The novel bonding motif is reminiscent of charge density waves in solid materials as all intermediate stages between delocalized to localized excess electrons would be covered. However, for the localized scenario there appears to be a closer analogy to the “Polyhedral Skeletal Electron Pair Theory” describing the bonding in the so-called Wade–Mingos molecular clusters.^27,28^ In this sense the excess electron pair would occupy the lowest bonding skeleton MO of the embedded silver clusters.

## Author contributions

M. J. drafted the reply, all authors discussed and finalised the manuscript.

## Conflicts of interest

There are no conflicts to declare.

## Supplementary Material
